# Effectiveness of a home-based telerehabilitation system in patients after total hip arthroplasty: study protocol of a randomized controlled trial

**DOI:** 10.1186/s13063-020-04791-4

**Published:** 2020-10-14

**Authors:** Chiara Busso, Gabriele Castorina, Marco Di Monaco, Daniel Rodriguez, Hadis Mahdavi, Simone Balocco, Marco Trucco, Marco Conti, Alessandro Castagna, Marco Alessandro Minetto

**Affiliations:** 1 Division of Physical Medicine and Rehabilitation, Department of Surgical Sciences, University of Turin, C.so Dogliotti 14, 10126 Turin, Italy; 2 Division of Physical Medicine and Rehabilitation, Presidio Sanitario San Camillo, Fondazione Opera San Camillo, Turin, Italy; 3 DyCare – Bio-Sensing Solutions S.L., Barcelona, Spain; 4 Department of Mathematics and Informatics, University of Barcelona, Barcelona, Spain; 5 MediSport, Human Performance Lab - Como and Varese, Varese, Italy; 6 IRCCS Humanitas Institute, Milan, Italy

**Keywords:** Hip dysfunction and Osteoarthritis Outcome Scale, Hip range of motion, Muscle strength, Timed Up-and-Go test, Total hip arthroplasty

## Abstract

**Background:**

The demand for total hip arthroplasty (THA) is quickly rising given the escalating global incidence of hip osteoarthritis, and it is widely accepted that the post-surgery rehabilitation is key to optimize outcomes. The overall objective of this study is to evaluate the effectiveness of a new telerehabilitation solution, ReHub, for the physical function and clinical outcome improvement following THA. The specific aims of this manuscript are to describe the study design, protocol, content of interventions, and primary and secondary outcomes and to discuss the clinical rehabilitation impact of the expected experimental results.

**Methods/design:**

This prospective, randomized, controlled, parallel-group trial will include 56 patients who had undergone primary THA. Patients are randomized to a control group (standard rehabilitation during the 2-week stay in the rehabilitation clinic followed by 3 weeks of unsupervised home-based rehabilitation) or an experimental group (standard rehabilitation during the 2-week stay in the rehabilitation clinic followed by 3 weeks of home-based ReHub-assisted telerehabilitation). The primary outcome is physical performance assessed through the Timed Up-and-Go (TUG) test. Secondary outcomes include independence level, pain intensity, hip disability, hip range of motion, muscle strength, and patient’s perception of clinical improvement.

**Discussion:**

Proving the clinical and cost-effectiveness of a home-based telerehabilitation program for physical and muscle function following THA could support its systematic incorporation in post-surgical rehabilitation protocols, which should be tailored to the individual and collective needs.

**Trial registration:**

ClinicalTrial.gov NCT04176315. Registered on 22 November 2019

## Background

The demand for total hip arthroplasty (THA) is quickly rising given the escalating global incidence of hip osteoarthritis [1, 2] that is related both to the aging population and to the increases in obesity and sedentary behavior [2, 3]. The efficacy of THA is well documented, and it is widely accepted that the post-surgery rehabilitation is key to optimize outcomes [4, 5], in particular when the rehabilitation is based on intensive and early progressive exercises [6], which lead to improved clinical outcomes and patient satisfaction, and reduction of complications and expenses. In the face of rapidly increasing health care costs, ensuring widespread cost-effective rehabilitation became a priority.

In recent years, novel telerehabilitation solutions (i.e., rehabilitation services delivered at home from a remote location through a telecommunication system and information technology) [7, 8] have been developed that allow professionals to remotely monitor rehabilitation programs, thereby improving patient adherence to rehabilitation programs and reducing the healthcare costs. Recent studies have provided preliminary evidences that a telerehabilitation program is associated with better clinical outcomes than conventional rehabilitation after THA. In fact, Karlon et al. [9] found that a 6-week telerehabilitation program based on video clips of common exercises (3 sessions/week) added to physical therapy sessions (3 sessions/week) was more effective for the recovery of physical function compared to conventional rehabilitation (physical therapy sessions plus home-based booklet-guided rehabilitation program). In a very recent study, Dias Correia et al. [10] also showed that an 8-week telerehabilitation program (30 min/day of exercise performed with a digital biofeedback system, 5–7 sessions/week) was associated with better outcomes than conventional rehabilitation (1 h/day of a home-based program provided by a physiotherapist, 3 sessions/week).

The usual rehabilitation settings and care path after THA in Italy consist of a short (2–3 weeks) stay in a rehabilitation clinic followed by home-based rehabilitation. The unsupervised execution of a home-based rehabilitation program implies that patients are individually charged with the responsibility to undertake exercises at a time and a place convenient to their needs and daily living schedules. However, low personal motivation and misunderstanding the instructions to execute the program can negatively affect the outcome of individual rehabilitation programs. Therefore, it has been suggested that patient supervision may be a key factor to achieve the best possible rehabilitation results in some populations [11].

We hypothesized that a home-based telerehabilitation program performed through the remote supervision of the patient’s performance and adherence can improve clinical outcomes compared to conventional (unsupervised) home-based care.

Therefore, the overall objective of this study is to evaluate the effectiveness of a new telerehabilitation solution, ReHub, for the physical function and clinical outcome improvement following THA. The specific aims of this manuscript are to describe the study design, protocol, content of interventions, and primary and secondary outcomes and to discuss the clinical rehabilitation impact of the expected experimental results.

## Methods

### Study design and randomization

**Fig. 1:**
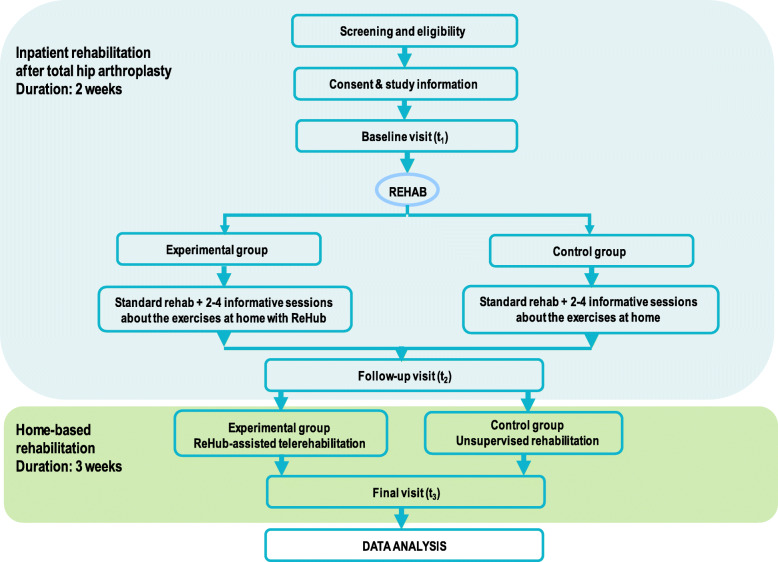
Study design diagram

The study is a prospective, randomized, controlled, parallel-group, open-label with blinded assessor trial that is conducted according to the SPIRIT recommendations [12]. Following informed consent, patients are randomized (with a 1:1 allocation ratio) to a control group or an experimental group (Fig. 1). Computer-generated randomization lists are used (using the website www.random.org) to sequentially distribute the patients into one of the two groups. Generation of the allocation sequence and assignment of participants to interventions are performed by one of the authors (MDM).

The study conforms to the guidelines of the Declaration of Helsinki, was approved by the local ethics committee (“Comitato Etico Interaziendale AOU Città della Salute e della Scienza di Torino”: protocol n. 0107065), and registered at the ClinicalTrial.gov website (identifier NCT04176315).

### Study setting and patients

The study setting is a rehabilitation clinic where patients of both genders who had undergone primary THA surgery are recruited. The inclusion criteria are as follows: (i) ability to understand and accept the study procedures and to sign an informed consent form, (ii) good familiarity with the Italian language, (iii) good predisposition to the use of technology or availability of a caregiver providing technological support to the patient, and (iv) availability to move to the rehabilitation clinic for a final visit. The exclusion criteria are as follows: (i) age < 60 or > 80 years and body mass index > 35 kg/m^2^; (ii) admission after THA revision surgery; (iii) contralateral hip osteoarthritis severely limiting patient mobility and ability to comply with a rehabilitation program; (iv) aphasia, dementia, or psychiatric comorbidity interfering with communication or adherence to the rehabilitation process; (v) respiratory, cardiac, metabolic, or other conditions limiting patient mobility and ability to comply with a rehabilitation program; and (vi) major medical complications occurring after surgery that prevented the discharge of the patient within 10 days after the surgery.

#### Interventions

Patients in both groups receive a standard rehabilitation protocol during the 2-week stay in the rehabilitation clinic, as directed by the medical staff, aimed to improve the hip range of motion (ROM), the global neuromuscular performance (walking, chair rise, etc.), and the strength of the hip and lower leg muscles. The program consists of 3 h/day of physiotherapy during weekdays (between Monday and Friday) and 1 h of activity on Saturday for a total of 16 h of activity per week.

In addition, patients in the experimental group perform also 1–2 sessions per week (2–4 sessions in total) of supervised familiarization with the ReHub telerehabilitation system (see below). Patients in the control group perform 1–2 sessions per week (2–4 sessions in total) of supervised familiarization with a printed guide describing the exercises to be performed at home (Fig. 1).

After discharge from the rehabilitation clinic, patients in the control group follow the standard home-based care (they receive a guide to performing the exercises, and they are asked to fill out a diary questionnaire designed to assess the adherence to the rehabilitation program), while patients in the experimental group follow a home-based telerehabilitation and telemonitoring program by using ReHub.

The home-based rehabilitation program consists in the execution (unsupervised for the control patients, ReHub-assisted for the experimental patients) of the following 5 exercises: (1) hip flexors, with a lateral support, flexion of the hip joint up to a maximum of 90° of ROM while bending the knee; (2) quadriceps (eccentric contraction), with a front support, lunge with both legs while bending the knees; (3) hip abductors, with a front support, abduction of the hip joint (with both knees extended) until the maximum ROM is allowed; (4) hip extensors, with a front support, extension of the hip joint until the maximum ROM is allowed; and (5) sit-to-stand, standing up from a chair and sitting down on it sequentially, with one foot slightly in front of the other and without the support of the upper limbs (arms are crossed at the wrists and held against the chest).

Patients are asked to perform the rehabilitation protocol according to the following workload: (i) daily execution of a single exercise session for 3 weeks, (ii) three series of ten repetitions for each exercise, and (iii) bilateral execution of exercises 1-3-4.

Concomitant care permitted during the home-based rehabilitation program includes the use of analgesics, when needed.

The criteria for discontinuing interventions include participant requests and intolerance to the rehabilitation program.

Retention of study participants is performed according to the following strategies: use of a systematic method for patient contact and appointment scheduling, study reminders, and emphasizing study benefits.

Provisions for post-trial care will consist of the standard care within the National Health Service.

#### Adverse events reporting and harms

Any unfavorable and unintended sign, symptom, or illness that develops or worsens during the period of the study is classified as an adverse event, whether or not it is considered to be related to the study treatment. Adverse events may include an exacerbation of a pre-existing illness, a condition that is detected after trial intervention administration, and continuous persistent disease or a symptom present at baseline that worsens following the administration of the trial treatment—and may be expected or unexpected. The number (events and individuals) and the nature of all adverse events reported to blind and unblind members of the research team are recorded. The period for adverse event reporting is following the signing of the study consent form until the last follow-up assessment. All adverse events are recorded and reviewed by the chief investigator (MAM).

#### Blinding and outcomes

**Fig. 2:**
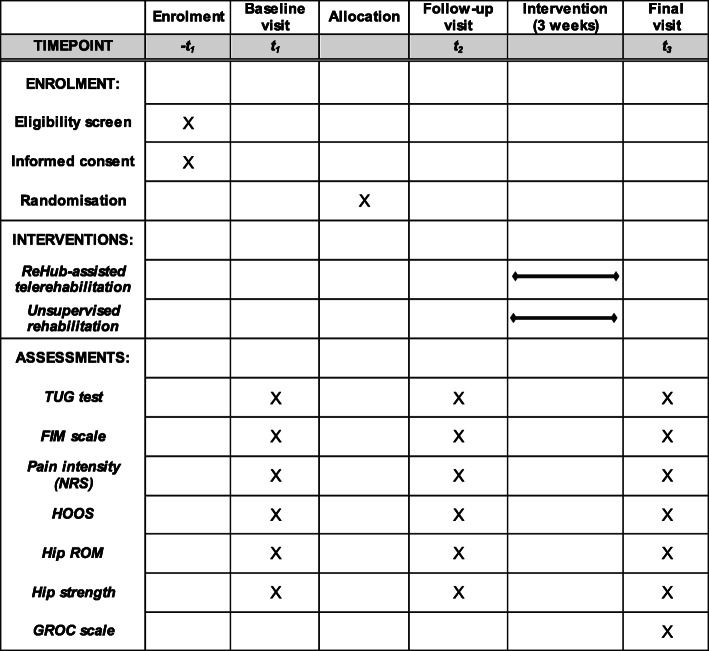
Standard Protocol Items: Recommendations for Interventional Trials (SPIRIT) figure showing the schedule of enrollment, interventions, and assessments

A blinded physician (GC) completes all functional assessments and gathers all clinical data on the electronic medical record of each patient, as reported in Fig. 2.

The following clinical data are acquired from electronic patient files and clinical assessments: gender, age, body mass index, and length of hospital stay.

The primary outcome is physical performance assessed through the Timed Up-and-Go (TUG) test that measures the time required for standing up from a chair, walking straight for 3 m, turning, walking back to the chair, and sitting down [13].

Secondary outcomes include the following: (i) independence level (assessed through the Functional Independence Measure (FIM) scale) [14]; (ii) resting and movement pain intensity (assessed through a Numerical Rating Scale with 0 corresponding to “no pain” and 100 corresponding to the “worst imaginable pain”)—resting pain intensity is measured prior to any study procedures, while movement pain is measured during active hip flexion and extension; (iii) hip disability (assessed through the Hip dysfunction and Osteoarthritis Outcome Scale (HOOS)) [15]; (iv) hip ROM and muscle strength (see below); (v) patient’s perception of clinical improvement (assessed through a 6-point Likert scale investigating the global rating of change (GROC): 1 = much worse, 2 = worse, 3 = same, 4 = improved, 5 = much improved, and 6 = completely recovered).

All outcomes are assessed at three points [see Figs. 1 and 2—baseline visit (*t*_1_), rehabilitation clinic admission; follow-up visit (*t*_2_), rehabilitation clinic discharge; final visit (*t*_3_), 3 weeks after discharge], with the exception of the GROC score that is assessed at the final visit only.

#### Assessment of hip ROM and muscle strength

All ROM tests are conducted (using a manual, plastic, 2-arm goniometer with 1° increments) by a single physiotherapist, blinded to the allocation group. Only the passive ROM of the operated side is assessed in the following order: flexion, extension, and abduction. Hip flexion and abduction ROM are measured with participants in the supine position, while hip extension ROM is measured in the side-lying position.

The maximal isometric voluntary contraction of the operated side is assessed using a wall-fixed dynamometer (model HCB 99 K50, Kern & Sohn, Balingen, Germany). Muscle strength tests are performed in the following order: knee extension, hip extension, and hip abduction. Knee extension is assessed with participants in the sitting position, with both hip and knee at 90° flexion. Hip extension and hip abduction are measured with participants in the standing position, with the body weight supported by the contralateral lower limb, hip in the neutral position, and knee in the extended position.

#### ReHub telerehabilitation system

ReHub is a digital platform for physical rehabilitation that offers personalized design and monitoring of therapeutic exercise programs to recover the functional capacity of the musculoskeletal system. The solution is composed of two main pillars: a cloud platform and a sensorized exercise kit. The cloud platform establishes effective communication between the patient and the healthcare professionals in charge of their rehabilitation. It allows physical therapists to create a rehabilitation program specifically tailored to each patient’s condition. Patients use the cloud platform to perform the exercises in their rehabilitation program with the help of DyCare’s proprietary wearable sensor that captures 3D motion data. The sensor is integrated into different exercise tools, although only a body strap is used in this study as the selected exercises do not require additional tools. The sensor records biomechanical parameters (such as ROM and movement speed) in real-time when used on the indicated body part while exercising. When patients do their prescribed exercises at home, intelligent algorithms deliver real-time biofeedback through a user interface and a virtual coach. The results can be viewed by the physical therapist to follow the progress of the patient, adapt the program remotely if needed, or chat with the patient by online messaging module through the platform.

#### Sample size estimation and statistical analyses

To elucidate a difference in physical performance between the intervention and the control groups, we used data from previous studies (average ± standard deviation of the TUG score in patients discharged home after post-surgery rehabilitation of 13 ± 3.5 s and TUG minimal detectable change of 2.5 s) [16, 17] to determine the required sample size. Twenty-five participants per group will provide adequate power to detect statistically significant differences in the TUG score between the two groups (allocation ratio 1; statistical power 80%; alpha level 0.05), but 56 patients will be recruited in total anticipating a dropout rate of 10%.

The statistical analysis of the results will be performed by a blinded expert. The Kolmogorov-Smirnov test will be adopted to check the normality of data distribution. Within- and between-group comparisons will be performed by a two-sample *t* test or one-way and two-way ANOVA.

The extent of missing data will be explored in the outcomes, especially the primary outcome. Patterns of missing data will be explored and predictors of missingness examined, especially if these vary by intervention. If necessary, multiple imputation will be used to impute missing data assuming the missingness mechanism is missing at random. A detailed statistical analysis plan will be agreed to before the end of data entry and before the treatment code is broken.

Data will be expressed as mean ± standard deviation (normally distributed data) or median and interquartile range (non-normally distributed data). The threshold for statistical significance will be set to *P* = 0.05. All statistical tests will be performed with MATLAB (The MathWorks, Inc., Natick, MA, USA) software package.

## Discussion

The study aims to assess the effectiveness of a home-based telerehabilitation program for the physical function and clinical outcome improvement following THA.

The randomized controlled design, blinding of the physician performing the outcome assessments, and use of valid tools for the assessment of physical performance and muscle strength (TUG score and dynamometer-based measurements of muscle strength under isometric conditions, respectively) are the notable strengths of the study.

Limitations of the study include the absence of specific investigations (e.g., electromyography, muscle ultrasonography) providing possible insights into the neural and muscular mechanisms underlying the effects of telerehabilitation in responder patients (or explaining the non-responder phenotype) and the lack of assessment of patient satisfaction and preferences providing possible insights into the treatment attributes considered most important by the patients [18].

Other study limitations are represented by the short intervention duration (3 weeks) and the lack of a long-term follow-up that could help determine whether the possible improvements in physical function induced by the telerehabilitation program may produce long-lasting benefits in physical performance and health.

Notwithstanding these limitations, demonstrating the effectiveness of a home-based telerehabilitation program for physical function and/or muscle strength improvement following THA could have relevant implications for the post-surgical rehabilitation process. In fact, telerehabilitation solutions can facilitate access and adherence to health interventions, reduce health care costs (associated with supervision, facility provision, and transport of patients), and contribute also to social distancing when it becomes necessary as an infection control action.

In addition, the direct and indirect costs of using the telerehabilitation system compared with the costs of the traditional rehabilitation programs commonly adopted in THA patients will also be assessed.

Therefore, proving the clinical and cost-effectiveness of a home-based telerehabilitation program for physical and muscle function following THA could support its systematic incorporation in post-surgical rehabilitation protocols, which should be tailored to the individual and collective needs.

Trial findings will be disseminated to the scientific community through publications and national and international conferences (authorship and time scales will be agreed by the Trial Management Group consisting of the trial manager, administrative staff, chief investigator, and statistician). Findings will also be disseminated to participants and patient organizations through social media and presentations at local conferences.

## Trial status

The Trial Management Group meets on a weekly basis to discuss progress and monitor recruitment, data returns, and so on.

The Trial Management Group meets also every 6 months for auditing trial conduct.

The first study participants were recruited into the trial in December 2019 (study protocol version 1, dated 20 September 2019). Patient recruitment and data collection are ongoing and will continue until the required number of study participants will be achieved (estimated date of the recruitment completion: November 2020).

## Data Availability

Data will be made available upon request to the corresponding author (Marco A. Minetto: marco.minetto@unito.it).

## Abbreviations

FIM: Functional Independence Measure
GROC: Global rating of change
HOOS: Hip dysfunction and Osteoarthritis Outcome Scale
ROM: Range of motion
THA: Total hip arthroplasty
TUG: Timed Up-and-Go
